# Establishing a primary care audit and feedback implementation laboratory: a consensus study

**DOI:** 10.1186/s43058-020-00103-8

**Published:** 2021-01-07

**Authors:** Sarah L. Alderson, Alexander Bald, Paul Carder, Amanda Farrin, Robbie Foy

**Affiliations:** 1 Leeds Institute of Health Science, University of Leeds, Leeds, UK; 2 School of Medicine, University of Leeds, Leeds, UK; 3 West Yorkshire Research and Development, NHS Bradford District and Craven Clinical Commissioning Group, Bradford, UK; 4 Leeds Institute of Clinical Trials Research, University of Leeds, Leeds, UK

**Keywords:** Primary healthcare, Inappropriate prescribing, Formative feedback, Implementation science, Clinical trial

## Abstract

**Background:**

There is a significant variation among individual primary care providers in prescribing of potentially problematic, low-value medicines which cause avoidable patient harm. Audit and feedback is generally effective at improving prescribing. However, progress has been hindered by research waste, leading to unanswered questions about how to include audit and feedback for specific problems and circumstances. Trials of different ways of providing audit and feedback in implementation laboratories have been proposed as a way of improving population healthcare while generating robust evidence on feedback effects. However, there is limited experience in their design and delivery.

**Aim:**

To explore priorities, feasibility, and ethical challenges of establishing a primary care prescribing audit and feedback implementation laboratory.

**Design and setting:**

Two-stage Delphi consensus process involving primary care pharmacy leads, audit and feedback researchers, and patient and public.

**Method:**

Participants initially scored statements relating to priorities, feasibility, and ethical considerations for an implementation laboratory. These covered current feedback practice, priority topics for feedback, usefulness of feedback in improving prescribing and different types of prescribing data, acceptability and desirability of different organization levels of randomization, options for trial consent, different methods of delivering feedback, and interest in finding out how effective different ways of presenting feedback would be. After receiving collated results, participants then scored the items again. The consensus was defined using the GRADE criteria. The results were analyzed by group and overall score.

**Results:**

Fourteen participants reached consensus for 38 out of 55 statements. Addressing antibiotic and opioid prescribing emerged as the highest priorities for action. The panel supported statements around addressing high-priority prescribing issues, taking an “opt-out” approach to practice consent if waiving consent was not permitted, and randomizing at lower rather than higher organizational levels. Participants supported patient-level prescribing data and further research evaluating most of the different feedback methods we presented them with.

**Conclusions:**

There is a good level of support for evaluating a wide range of potential enhancements to improve the effects of feedback on prescribing. The successful design and delivery of a primary care audit and feedback implementation laboratory depend on identifying shared priorities and addressing practical and ethical considerations.

**Supplementary Information:**

The online version contains supplementary material available at 10.1186/s43058-020-00103-8.

Contributions to the literature

- There is significant variation among individual primary care providers in prescribing of potentially harmful, problematic medicines.
- Audit and feedback appears effective at improving prescribing in primary care; however, there is limited knowledge on how to improve its effectiveness.
- A primary care prescribing implementation laboratory has been suggested as a potential solution with opportunities for researchers and healthcare systems to conduct embedded collaborative research to increase impact and effectiveness.
- Expert recommendations on how to optimize the design of a primary care prescribing audit and feedback implementation laboratory include addressing high-priority prescribing issues, opt-out consent for practices, access to detailed prescribing data, and randomization at lower rather than higher organizational levels.
- Establishing a primary care prescribing implementation laboratory depends on identifying shared priorities and addressing practical and ethical considerations.

## Introduction

There is a significant variation among primary care providers in potentially harmful and relatively ineffective prescribing [1–4]. Prescribing of medicines such as antibiotics, opioids, anticholinergics, and non-steroidal anti-inflammatory drugs (NSAIDs) is associated with wide-ranging harms, including increased mortality, hospitalization, falls, dependence, and antimicrobial resistance [3, 5, 6]. It also represents poor value for money in resource-constrained healthcare systems [7].

A range of approaches has been used to address problematic prescribing, including financial incentives, electronic decision support tools, and educational outreach [1, 8, 9]. Audit and feedback is often used as a core component of these approaches; it aims to improve patient care through collecting and feeding back performance data to providers [10]. Feedback may improve prescribing in several ways, including alerting providers to the need for action, encouraging providers to think twice before initiating medicines, and triggering structured reviews to rationalize medicines for individual patients [1]. Audit and feedback offers the relative efficiencies of harnessing routine electronic health record data, often available for prescribing [11] and having a wide population reach. High-performing health systems tend to feature audit and feedback as an evidence-based, scalable, and relatively inexpensive strategy to encourage uptake of best practice [12].

Audit and feedback is generally modestly effective. A Cochrane review of 140 randomized trials found that audit and feedback produced a median of 4.3% absolute improvement in processes of care, such as prescribing [10]. However, there is a wide variation in effectiveness. A quarter of audit and feedback interventions had relatively large, positive effects of up to 16% while another quarter had no or even negative effects. Feedback effects are generally greater when the source is a supervisor or colleague, it is provided more than once, it is delivered in both verbal and written formats, and it includes both explicit targets for change and action plans. However, there are still many uncertainties about how to optimize audit and feedback [13]. Progress has been hindered by research waste in this field through a lack of cumulative learning from duplicative trials of audit and feedback [14]. This means that those responsible for designing and delivering quality improvement programs will continue to have unanswered questions about how to include audit and feedback for specific problems and circumstances [14].

To understand when audit and feedback is most likely to be effective and how to optimize it, we need to move away from two-arm trials of audit and feedback compared with control in favor of head-to-head trials of different ways of providing audit and feedback. Implementation laboratories use a learning health system approach, involving collaborations between healthcare organizations providing audit and feedback at scale, and researchers, to embed head-to-head trials into quality improvement programs. Implementation laboratories have been proposed to improve the impact of audit and feedback while simultaneously producing generalizable knowledge about how to optimize the effects of such quality improvement initiatives [15]. A “radical incrementalism” approach involves making sequential, small changes, supported by tightly focused evaluations to cumulatively improve outcomes. It is already used in public policy and in business. For example, Amazon and Google randomize potential customers to different presentations of their products online to understand what drives purchases. It is also highly applicable to healthcare [16]. While trials of different feedback methods have been embedded within existing audit programs [17], there is still limited experience in establishing audit and feedback implementation laboratories.

Our earlier work involved interviewing primary care staff (physicians, nurses, and managers) about a range of evidence-based recommendations and involved primary care physicians in developing an implementation package that included audit and feedback [18]. The intervention package successfully reduced high-risk prescribing in primary care [19]. Interviews with primary care physicians and prescribing data identified opioid prescribing as a high-priority topic for improvement [4, 20]. We adapted this feedback intervention and delivered a 1-year feedback campaign on opioid prescribing in primary care. Our controlled interrupted time-series analysis found that the number of adults prescribed any opioid (excluding those with coded cancer diagnoses) fell during the intervention year [Alderson SL, Farragher TM, Willis TA, Carder P, Johnson S, Foy R: The effects of an evidence and theory-informed feedback intervention on opioid prescribing for non-cancer pain in primary care: a controlled interrupted time series analysis. Submitted.]. We interviewed physicians and found our feedback campaign was well-received by practices and worked by alerting prescribers to the need for action, prompting prescribers to think twice before initiating opioids, and encouraging medication reviews of patients who may not benefit from opioids [Wood S, Foy R, Willis TA, Carder P, Johnson S, Alderson SL: General practice responses to opioid prescribing feedback: a qualitative process evaluation. Submitted.]. We also identified remediable weaknesses.

We are planning an audit and feedback implementation laboratory to address high-priority prescribing issues in primary care in the UK. Our overall program of work is strongly embedded in the primary care physicians’ perspectives and experiences. In this study, we sought wider service, research and public perspectives on prescribing priorities, and the feasibility and ethical challenges of establishing an implementation laboratory [21].

## Methods

We used a two-stage Delphi consensus development process [22] to define key features of the planned implementation laboratory. The Delphi method is an iterative process which involves collecting, analyzing, and sharing the anonymous opinions of key stakeholders. It is useful when there is incomplete knowledge about a problem, particularly when the goals are to improve understanding and suggest solutions [22].

We identified potential participants as local primary care pharmacy leads responsible for medicine optimization in primary care, international audit and feedback researchers (affiliated to the Audit and Feedback Metalab [19]), and an existing primary care Patient and Public Involvement and Engagement (PPIE) panel with prior experience in implementation research [23]. This would ensure that our Delphi process would account for service, research, and public perspectives. We planned for 15–30 panelists in considering likely reliability and feasibility [22]. There is no agreement about the optimal size of a Delphi panel, with many including under 20 people [24, 25].

Using online surveys [26], we presented participants with questions and statements around priorities for an implementation laboratory and feasibility and ethical considerations (Additional file 1). These covered the following:

- Current feedback practice—how feedback on prescribing is currently delivered to primary care practices (for medicine optimization leads only)
- Priority topics for feedback—offering six options of antibiotic prescribing, opioids for chronic non-cancer pain, gabapentinoids, anticholinergic burden, prescribing safety indicators, and prescribing in low kidney function
- Perceived usefulness of audit and feedback in improving prescribing and importance of improving its effectiveness
- Perceived usefulness of different types of prescribing data, such as total numbers of prescriptions, numbers of patients prescribed medicines, and numbers of patients in high-risk subgroups prescribed medicines
- Acceptability of different levels of randomization—at four levels of practices, practice networks (small groups of practices covering 50,000 patients), clinical commissioning group (larger area-wide groups of practices with responsibility for commissioning healthcare), and sustainability and transformation plan (44 larger geographical areas covering NHS England, now referred to as integrated care systems)
- Acceptability and desirability of options for trial consent—including “opt-in” consent for primary care practices, “opt-out” consent for practices (so that they would be included in any trial by default unless they actively decline), consent at higher organizational levels (clinical commissioning group or sustainability and transformation plan), and waiving of consent
- Acceptability and desirability of different methods of delivering feedback—such as paper-based hard copies, emailed PDFs, or online dashboards
- Interest in finding out how effective different ways of delivering feedback would be—such as using different comparators, visual presentations, and additional education outreach and training

We drew on previous work describing potential challenges in setting up implementation laboratories and team brainstorming [13, 15, 21]. We piloted the statements with two feedback researchers and three medicine optimization professionals. For most statements, participants could rate according to one or more of importance (potential to improve patient safety and care), priority (based on whether there are existing interventions to improve safety and care), usefulness (to primary care providers), acceptability, and desirability (for research purposes). Statements were rated on a 1–9 scale, where “1” indicated the strongest disagreement and “9” the strongest agreement. We also provided an “unable to score” response if participants felt unable to rate a statement. Participants could also comment on the rationale for their decisions. We asked participants to complete the survey independently in round 1. Support for a statement was defined as 70% or more of responses scoring ≥ 7 and 15% or less responses rating the outcome ≤ 3.

We removed statements rejected as “not important” for round 2 to maximize efficiency. The remaining statements were then fed back to all participants via email. We showed each participant their own scores from round 1, as well as the median scores from all participants and for each of the three stakeholder groups [22]. Participants then independently re-rated each statement [27]. We analyzed the final responses for all participants and by each stakeholder group.

The University of Leeds School of Medicine Research Ethics Committee approved the study (ref no: MREC 18-049).

## Results

**Fig. 1:**
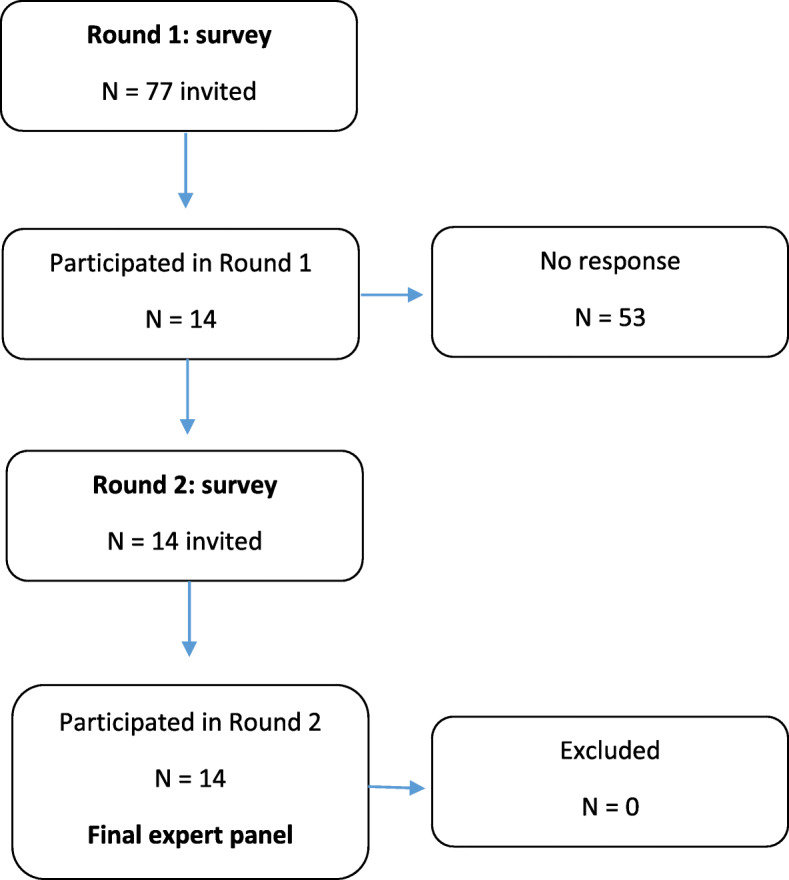
Flowchart of the consensus process

Fourteen participants comprised eight females and six males, five medicine optimization leads, five feedback researchers (two from The Netherlands, two from Canada, and one from the USA), and four PPIE panelists out of 15, 52, and 10 invited, respectively (Table 1). All completed rounds 1 and 2, with a 100% completion rate (Fig. 1).

**Table 1:** Characteristics of participants

Participant characteristics	Number (%)
**Gender**	
**Female**	8 (57)
**Male**	6 (43)
**Nation**	
**UK**	9 (64)
**The Netherlands**	2 (14)
**Canada**	2 (14)
**USA**	1 (7)
**Role**	
**Audit and feedback researcher**	5 (36)
**Medicine optimization lead**	5 (36)
**Patient and public involvement and engagement**	4 (28)

The medicine optimization leads reported considerable variability in the current prescribing feedback practice for primary care (Additional file 2). While all reported using a comparator, none reported using an online dashboard (linked or not to electronic health records), posting paper reports, nor initially providing intense feedback before reducing frequency.

Eighteen out of 55 statements were supported in round 1 (Additional file 3). All 55 statements were taken forward to round 2 as no statement reached the threshold for rejection. Thirty-eight statements were supported in round 2 (Table 2), while eleven had no clear support.

**Table 2:** Recommendations for a primary care prescribing implementation laboratory

No.	Recommendation	A&F researcher’s (*n* = 5) score (1–9)	Medicine optimization lead’s (*n* = 5) score (1–9)	Patient and public involvement and engagement’s (*n* = 4) score (1–9)	% Consensus (*n* = 14)
Prescribing issues for A&F					
Importance					
1.	Antibiotic prescribing	9	9	8.5	93
2.	Prescribing safety indicators	7	8	7.5	93
3.	Opioid medication for chronic, non-cancer pain	9	9	7	86
4.	Anticholinergic burden	6	8	7	79
5.	Prescribing in low kidney function	8	7	6.5	72
6.	Gabapentin and pregabalin painkillers	5	8	6	72
Priority					
7.	Antibiotic prescribing	9	9	8.5	100
8.	Opioid medication for chronic, non-cancer pain	9	8.5	7.5	93
9.	Anticholinergic burden	7.5	7	7	86
10.	Prescribing safety indicators	7.5	7	7.5	79
11.	Prescribing in low kidney function	7.5	6	6.5	72
	*Gabapentin and pregabalin painkillers*	*6.5*	*9*	*8*	*67*
Audit and feedback as a method to improve primary care prescribing					
12.	Importance	8	8	9	93
13.	Usefulness	8	8	7	86
Usefulness of the types of data					
14.	Subgroups of patients at high risk of dose escalation or adverse effects	8	9	9	100
15.	Number of patients taking opioid medication, excluding patients with a palliative care diagnosis	6.5	8	7.5	86
16.	Number of patients taking opioid medication, excluding patients taking medication for drug addiction	6	8	8	73
	*Specific opioid medications*	*7*	*7*	*7*	*50*
	*Number of patients taking opioid medication*	*5*	*6*	*7.5*	*50*
	*Total number of opioid prescriptions*^a^	*3*	*3*	*3.5*	*7*
Randomization level					
17.	Randomization at the practice level	9	9	9	100
18.	Randomization at the primary care network level	9	9	8	91
19.	Randomization at the clinical commissioning group level	8	8	7.5	75
	*Randomization at the Sustainability and Transformation Plan level*	*9*	*7*	*8*	*63*
Consent					
Acceptable					
20.	Provide practices information on the trial and allow them to withdraw from the trial if they wish (practice opt-out)	9	9	9	100
	*Consent at the clinical commissioning group level for data access*	*6*	*8*	*7.5*	*68*
	*Waive consent as the burden of responding to consent request is higher than taking part in the trial*	*7*	*6*	*8*	*62*
	*Consent practices individually, asking them to sign up to an opioid prescribing feedback trial (practice opt-in)*	*7*	*5*	*6.5*	*36*
	*Consent at the Sustainability and Transformation Plan level for data access*	*6*	*6*	*6*	*36*
Ideal					
21.	Provide practices information on the trial and allow them to withdraw from the trial if they wish (practice opt-out)	8	8	8	86
22.	Waive consent as the burden of responding to consent request is higher than taking part in the trial	8	9	8	77
23.	Consent at the clinical commissioning group level for data access	7	8	4.5	77
	*Consent at the Sustainability and Transformation Plan level for data access*	*6*	*7*	*6.5*	*64*
	*Consent practices individually, asking them to sign up to an opioid prescribing feedback trial (practice opt-in)*	*5*	*2*	*5.5*	*14*
Feedback delivery method					
Acceptable					
24.	Have an online dashboard that practices can log into that connects to the EHR to identify patients where a review is needed	9	9	9	100
	*Have an online dashboard that practices can log into to view their report (not linked to the EHR system)*	*7*	*7*	*7.5*	*64*
	*Send a PDF copy of the report via email to each practice*	*6*	*6*	*6.5*	*43*
	*Provide (multiple) copies of a paper-based report to each practice*	*6*	*3*	*4*	*21*
Ideal					
25.	Have an online dashboard that practices can log into that connects to the EHR to identify patients where a review is needed	9	9	9	100
26.	Have an online dashboard that practices can log into to view their report (not linked to the EHR system)	8	7	7	79
	*Send a PDF copy of the report via email to each practice*	*5*	*5*	*5.5*	*7*
	*Provide (multiple) copies of a paper-based report to each practice*^a^	*3*	*1*	*2.5*	*2*
Feedback modifications to test for effectiveness					
27.	Whether feedback identifying specific behaviors to be changed is more effective	8	8	7.5	93
28.	Whether different comparators within the reports are more effective	8	8	7	86
29.	Whether feedback about an individual or aggregated cases is more effective	7	9	7.5	79
30.	Whether the frequency or the number of times feedback is delivered affects achievement	6	7	7	79
31.	Whether different visual interpretations of the data are more effective	7	9	7.5	71
32.	Whether feedback on its own is more (cost-) effective than feedback delivered with educational outreach or training	8	8	7	71
33.	Whether different delivery methods of providing feedback are more effective	7	7	8	71
	*Whether asking practitioners to document the implications of changing practice is more effective*	*6*	*7*	*5.5*	*43*
Involved in designing feedback reports					
34.	General practitioners	8	9	9	100
35.	Primary care pharmacists	8	8	9	93
36.	Medicine optimization leads	8	9	8.5	92
	*Clinical commissioners*	*6*	*6*	*7*	*46*
	*Patient and public involvement experts*	*5*	*6*	*6*	*36*

Italicized text indicates areas not reaching consensus

^a^Areas reaching consensus not for inclusion

### Prescribing priorities

All prescribing options, except gabapentinoids, were rated as important, and priorities for improvement, with antibiotics and opioids for chronic, non-cancer pain, rated as the highest priority (100% and 93%, respectively). All panelists agreed that feedback interventions were both useful (86%) and important (93%) as a method to improve primary care prescribing.

### Ethical issues

The panel agreed including all primary care practices unless they actively withdrew from the implementation laboratory (opt-out) was both the most acceptable (100%) and desirable (86%) approach to consent. Individual practice consent (opt-in) was considered neither acceptable (36%) nor desirable (14%). Although waiving consent was agreed as desirable for recruitment (77%), the medicine optimization leads were uncertain about acceptability (62%). Consent at higher organizational levels (clinical commissioning group or Sustainability and Transformation Plan) for data access was less acceptable (68%; 36%) or desirable (77%; 64%).

The majority agreed that primary care providers (100%), primary care pharmacists (93%), and medicine optimization leads (92%) should be involved in designing feedback reports to improve primary care prescribing. The panelists considered that clinical commissioning (46%) and PPIE involvement (36%) were less useful.

### Feasibility issues

The panel considered the feedback of specific data, such as subgroups of high-risk patients (100%) or excluding patients not targeted by the feedback (86%), as the most useful for changing clinical practice. Less specific data, such as the total number of prescriptions of a medication, was not considered useful (7%).

For an implementation laboratory, randomization levels at the local level (practice or primary care network) were the most ideal (100%; 91%). Randomization by larger areas such as Sustainability and Transformation Plan level was not ideal (63%).

Online dashboards that connected to the electronic health record system were both acceptable (100%) and desirable (100%); dashboards unconnected to the electronic health record system received less support (64%; 79%). Providing multiple paper copies or emailing PDFs of feedback reports were considered neither desirable (2%; 7%) nor acceptable (21%; 43%).

Seven out of eight suggested methods of presenting feedback were agreed as priorities for the implementation laboratory for further effectiveness evaluations. The statements “whether feedback identifying specific behaviors to be changed is more effective” (93%) and “whether different comparators within the reports are more effective” (79%) received the strongest support. Determining “whether asking practitioners to document the implications of changing practice is more effective” was not recommended for testing (43%).

## Discussion

We have identified prescribing priorities and elicited key ethical and feasibility considerations to guide the establishment of a primary care prescribing implementation laboratory. All participants agreed that audit and feedback is both useful and important as a method to improve primary care prescribing. There is a notable support to evaluate a wide range of potential enhancements to improve the effects of feedback on prescribing, which collectively could be addressed by sequential trials within an implementation laboratory.

The panel supported action on a range of problems, prioritizing reductions in prescribing of antibiotics and opioids for chronic, non-cancer pain. Both are recognized internationally as public health threats, with respective legacies of antibiotic resistance and dependence [4, 28]. Other problems deemed as highly important, such as prescribing safety indicators, were rated lower as priorities, given existing quality improvement work.

Detailed prescribing data was preferred, specifically patients at high risks of dose escalation and adverse effects, and excluding those patients not targeted by the feedback [20]. Extracting this data requires access to electronic health record systems and data sharing agreements, thereby increasing complexity and hurdles compared to using current freely available English NHS prescribing data such as OpenPrescribing.com [29] and ePACT2 [30]. These initiatives and others providing practice-aggregated prescriptions by medicine category were not helpful for feedback. Medicines with more than one indication may be less suitable for feeding back the total number of prescriptions as exceptions are easier to justify [31]. Highlighting smaller high-priority subgroups of patients may avoid overwhelming practices, causing avoidance and inaction, than all patients prescribed antibiotics or opioids.

Consenting practices through an opt-out approach was agreed to be the most acceptable and desirable. Clinical commissioning group consent or waiving consent completely was desirable, but less acceptable. Waiving consent to reduce participation burden and undermining of results is complicated by the current data protection regulation but may increase participation of practices not routinely involved in primary care research and most likely to benefit. This method was recently used in a UK-wide audit and feedback trial to reduce antibiotic prescribing [32].

The panel recommended randomization in implementation laboratory trials should occur at the practice or primary care network level. Primary care network level may reduce trial arm contamination as practices increasingly work closer together, share staff, and develop local quality improvement initiatives [33]. Practice-level randomization may increase inherent risks of organizational instability for an implementation laboratory where sequential trials are likely to occur over a period of years.

The panel recommended feedback using online dashboards that connect to the electronic health record system, although not currently used by medicine optimization leads. Evidence suggest dashboards providing easily accessible and immediate access to information (e.g., as a screen saver) may improve adherence to recommendations and improve patient outcomes [34]. Multiple dashboards targeting different priorities may cause user fatigue, increase barriers to data access, and without regular prompts may reduce impact [34].

Nearly all suggestions for feedback modifications presented were recommended for further evaluation of effectiveness. This is not unexpected given the lack of progress in the understanding of audit and feedback mechanisms of actions or the key “active ingredients” to produce change [13].

There were three main study limitations. Firstly, Delphi consensus processes can produce but not guarantee the validity of an agreed set of endorsements. For our purposes, given uncertainties around the optimal design of an audit and feedback implementation laboratory, we leveraged a reasonably diverse range of stakeholder perspectives and attained consensus for most statements within two rounds, which lends legitimacy to our future plans.

Secondly, despite our best efforts, we did not achieve a larger sample size. Less than a fifth of people invited participated in the consensus process, raising the possibility that our respondents had unusually high interests in audit and feedback or research and those not participating felt unable to contribute expertise in this area. The lowest response was from the audit and feedback “Metalab” mailing list of researchers (10% of those invited). We surmise two possible reasons why this may be: Firstly, the mailing list includes audit and feedback researchers; however, it is also open to those interested in audit and feedback more generally (e.g., quality improvement staff) who may not have considered themselves to be experts and therefore did not feel the study was appropriate for them. Secondly, the researchers and experts on the mailing list have been collaborators in our previous and planned work and considered themselves insufficiently distanced. The response rates for medicine optimization leads and patients and public group (33% and 40%, respectively) are more representative of survey responses generally. We were unable to collect information on non-responders to determine non-responder bias. We at least avoided any potential attrition bias by ensuring complete participation in both rounds. Moreover, we still achieved a reasonable overall balance of participants.

Finally, we did not seek the views of primary care providers in this study, although such views were represented in earlier work and within the audit and feedback researchers, medicine optimization leads, and study team. We did gain international perspectives from audit and feedback researchers with emerging experience of developing implementation laboratories in other healthcare contexts. Our patient and public participants also had an experience of oversight for previous trials of audit and feedback in primary care [19] and helped ensure that plans for an implementation laboratory took account of public priorities. Future work should aim to involve end-users, including physicians, in the intervention and implementation laboratory design.

The key endorsements for developing a primary care prescribing implementation laboratory concern the need for specific prescribing data, offering feedback at the lowest aggregate level, using opt-out consent for practices (although preferably waived), and developing online dashboards connected to electronic health record systems. These endorsements fit with those suggested by Brehaut et al. to optimize the effectiveness of audit and feedback [35], although some received stronger support than others. Most methods of presenting feedback were recommended for effectiveness evaluation, reflecting continuing uncertainty on how best to optimize audit and feedback in routine service development. Different variants of audit and feedback, such as using an average- or high-performing comparator, have little or no cost implications. This suggests that marginal gains in feedback effects, such as an additional 1% in effectiveness (from 4 to 5%) in problematic prescribing, are likely to be worthwhile at a population level and would be relatively straightforward to test within an implementation laboratory [21].

## Conclusions

This small study has produced expert consensus-based endorsements for the optimal design of an audit and feedback implementation laboratory addressing high-priority prescribing issues in UK primary care. Any successful collaboration will depend on identifying shared priorities and addressing practical and ethical considerations. An implementation laboratory at scale may lead to the development of the first national clinical audit to implement radical incrementalism to improve effectiveness.

## Supplementary Information

**Additional file 1.** CROP Consensus Round 1

**Additional file 2. **Medicines Optimisation Lead’s (*n* = 5) current prescribing feedback practice

**Additional file 3.** Results from the first round of consensus survey. Results that changed in round 2 are in italics and bold

## Data Availability

The datasets generated and/or analyzed during the current study are not publicly available due to confidentiality but are available from the corresponding author on reasonable request.

## Abbreviations

PPIE: Patient and Public Involvement and Engagement

## References

[1] Guthrie B, Kavanagh K, Robertson C, Barnett K, Treweek S, Petrie D (2016). Data feedback and behavioural change intervention to improve primary care prescribing safety (EFIPPS): multicentre, three arm, cluster randomised controlled trial. Br Med J.

[2] MacBride-Stewart S, Marwick C, Houston N, Watt I, Patton A, Guthrie B (2017). Evaluation of a complex intervention to improve primary care prescribing: a phase IV segmented regression interrupted time series analysis. Br J Gen Pract.

[3] Pérez T, Moriarty F, Wallace E, McDowell R, Redmond P, Fahey T. Prevalence of potentially inappropriate prescribing in older people in primary care and its association with hospital admission: longitudinal study. BMJ. 2018;363:k4524. 10.1136/bmj.k4524 PMC6233705 30429122

[4] Foy R, Leaman B, McCrorie C, Petty D, House A, Bennett M, et al. Prescribed opioids in primary care: cross-sectional and longitudinal analyses of influence of patient and practice characteristics. BMJ Open. 2016;6(e010276). 10.1136/bmjopen-2015-010276 PMC4874107 27178970

[5] Ray WA, Chung CP, Murray KT, Hall K, Stein C (2016). Prescription of long-acting opioids and mortality in patients with chronic noncancer pain. JAMA..

[6] Li Y, Mölter A, White A, Welfare W, Palin V, Belmonte M (2019). Relationship between prescribing of antibiotics and other medicines in primary care: a cross-sectional study. Br J Gen Pract.

[7] Duerden M, Millson D, Avery A, Smart S. The quality of GP prescribing: The King’s Fund; 2011; London.

[8] Colquhoun HL, Squires JE, Kolehmainen N, Fraser C, Grimshaw JM (2017). Methods for designing interventions to change healthcare professionals’ behaviour: a systematic review. Implement Sci.

[9] Dreischulte T, Donnan P, Grant A, Hapca A, McCowan C, Guthrie B (2016). Safer prescribing—a trial of education, informatics, and financial incentives. N Engl J Med.

[10] Ivers N, Jamtvedt G, Flottorp S, Young JM, Odgaard-Jensen J, French SD, et al. Audit and feedback: effects on professional practice and healthcare outcomes. Cochrane Libr. 2012; Issue 6. Art. No.: CD000259. 10.1002/14651858.CD000259.pub3 PMC11338587 22696318

[11] Curtis HJ, Goldacre B (2018). OpenPrescribing: normalised data and software tool to research trends in English NHS primary care prescribing 1998–2016. BMJ Open.

[12] Hysong SJ, Best RG, Pugh JA (2006). Audit and feedback and clinical practice guideline adherence: making feedback actionable. Implement Sci.

[13] Ivers NM, Sales A, Colquhoun H, Michie S, Foy R, Francis JJ (2014). No more ‘business as usual’ with audit and feedback interventions: towards an agenda for a reinvigorated intervention. Implement Sci.

[14] Foy R, Eccles MP, Jamtvedt G, Young J, Grimshaw JM, Baker R. What do we know about how to do audit and feedback? Pitfalls in applying evidence from a systematic review. BMC Health Serv Res. 2005;5;50. 10.1186/1472-6963-5-50 PMC1183206 16011811

[15] Ivers NM, Grimshaw JM (2016). Reducing research waste with implementation laboratories. Lancet.

[16] Halpern D, Mason D (2015). Radical incrementalism. Evaluation..

[17] Hartley S, Foy R, Walwyn RE, Cicero R, Farrin AJ, Francis JJ (2017). The evaluation of enhanced feedback interventions to reduce unnecessary blood transfusions (AFFINITIE): protocol for two linked cluster randomised factorial controlled trials. Implement Sci.

[18] Glidewell L, Willis TA, Petty D, Lawton R, McEachan RRC, Ingleson E (2018). To what extent can behaviour change techniques be identified within an adaptable implementation package for primary care? A prospective directed content analysis. Implement Sci.

[19] Willis TA, Collinson M, Glidewell L, Farrin AJ, Holland M, Meads D (2020). An adaptable implementation package targeting evidence-based indicators in primary care: a pragmatic cluster-randomised evaluation. PLoS Med.

[20] McCrorie C, Closs SJ, House A, Petty D, Ziegler L, Glidewell L (2015). Understanding long-term opioid prescribing for non-cancer pain in primary care: a qualitative study. BMC Fam Pract.

[21] Grimshaw J, Ivers N, Linklater S, Foy R, Francis JJ, Gude WT, et al. Reinvigorating stagnant science: implementation laboratories and a meta-laboratory to efficiently advance the science of audit and feedback. BMJ Qual Saf. 2019;0:1–8. 10.1136/bmjqs-2018-008355 PMC6559780 30852557

[22] Murphy MK, Black NA, Lamping DL, McKee CM, Sanderson CF, Askham J (1998). Consensus development methods, and their use in clinical guideline development. Health Technol Assess (Winchester, England).

[23] Gray-Burrows KA, Willis TA, Foy R, Rathfelder M, Bland P, Chin A, et al. Role of patient and public involvement in implementation research: a consensus study. BMJ Qual Saf. 2018;27(10):858–6. 10.1136/bmjqs-2017-006954 PMC6166593 29666310

[24] Akins RB, Tolson H, Cole BR (2005). Stability of response characteristics of a Delphi panel: application of bootstrap data expansion. BMC Med Res Methodol.

[25] Diamond IR, Grant RC, Feldman BM, Pencharz PB, Ling SC, Moore AM (2014). Defining consensus: a systematic review recommends methodologic criteria for reporting of Delphi studies. J Clin Epidemiol.

[26] JISC. OnlineSurveys. Available from: https://www.onlinesurveys.ac.uk/. Accessed 10 Apr 2019.

[27] Brookes ST, Macefield RC, Williamson PR, McNair AG, Potter S, Blencowe NS (2016). Three nested randomized controlled trials of peer-only or multiple stakeholder group feedback within Delphi surveys during core outcome and information set development. Trials..

[28] Tacconelli E, Pezzani MD (2019). Public health burden of antimicrobial resistance in Europe. Lancet Infect Dis.

[29] EBM DataLab. OpenPrescribing.net: University of Oxford; 2017.

[30] NHS Business Services Authority. EPACT2. https://www.nhsbsa.nhs.uk/epact2. Accessed 16 Apr 2020.

[31] Improvement NEaN (2019). Items which should not routinely be prescribed in primary care: guidance for CCGs.

[32] Hallsworth M, Chadborn T, Sallis A, Sanders M, Berry D, Greaves F (2016). Provision of social norm feedback to high prescribers of antibiotics in general practice: a pragmatic national randomised controlled trial. Lancet.

[33] Baird B. Primary care networks explained: The King’s Fund; 2019; London.

[34] Dowding D, Randell R, Gardner P, Fitzpatrick G, Dykes P, Favela J (2015). Dashboards for improving patient care: review of the literature. Int J Med Inform.

[35] Brehaut JC, Colquhoun HL, Eva KW, Carroll K, Sales A, Michie S (2016). Practice feedback interventions: 15 suggestions for optimizing effectiveness. Ann Intern Med.

