# Supplementary material for: Establishing a primary care audit and feedback implementation laboratory: a consensus study
Source: Implement Sci Commun. 2021 Jan 7;2:3. doi: 10.1186/s43058-020-00103-8 (PMC7792204; doi:10.1186/s43058-020-00103-8)
Supplement: Supplementary file 1 — Additional file 1. CROP Consensus Round 1 [file 43058_2020_103_MOESM1_ESM.pdf]

Appendix 1:
CROP Consensus Round 1

Introduction

Audit and Feedback (A&F) are one of the methods that are routinely used to improve patient care. Health care professionals, working either in a team or individually, receive feedback on their performance by reflecting on data derived from their routine practice. A&F are intended to enhance professional performance and thereby improve the quality of health care and patient safety. We previously used A&F in the Campaign to Reduce Opioid Prescribing (CROP). CROP reduced predicted opioid prescription spending by £900,000 in West Yorkshire.

We are exploring the feasibility of scaling up the CROP intervention for a larger primary care randomised controlled trial (RCT) to reduce harmful opioid prescribing whilst improving the effectiveness of A&F.

In this first stage of the consensus process we will present you with a range of prescribing priority issues, ethical and governance issues and potential modifications to A&F reports that may increase effectiveness.

Please grade each item on the scale of 1 to 9 in terms of importance, priority and usefulness as prompted by each question. There is also an ‘Unable to score’ response if you are unable to rate any outcome. If you require more information to answer the question please click on 'more info' to see additional information.There will also be space for you to leave comments that you feel are important to consider in our interpretation of the results.

Please click next to move to the next page to give your consent to take part in this study.

Consent

Consent to take part in: OPTIMISING THE CONTENT, FORMAT AND DELIVERY OF THE CAMPAIGN TO REDUCE OPIOID PRESCRIBING FOR A NATIONAL RANDOMISED CONTROLLED TRIAL

Version 1.1 - 4th February 2019

I confirm that I have read and understand the information sheet v1.2 dated 9th April 2019 explaining this research project and I have had the opportunity to ask questions about the project. *(initial next to the statement if you agree)* ✱ *Required*

I understand that my participation is voluntary and that I am free to withdraw at any time during the consensus process and up to seven days after without giving any reason and without there being any negative consequences. Any data I have contributed to earlier rounds of the consensus process will continue to be used. To withdraw please contact Dr Sarah Alderson (email: s.l.alderson@leeds.ac.uk). (*Initial next to the statement if you agree)* ✱ *Required*

I understand that any information I provide, including personal details, will be confidential, stored securely and only accessed by those carrying out the study. I or my institution will not be identifiable in the report or reports that result from the research without my permission. (*Initial next to the statement if you agree)* ✱ *Required*

I agree for the data collected from me to be stored and used in relevant future research in an anonymised form. (*Initial next to the statement if you agree)* ✱ *Required*

I understand that other genuine researchers will have access to this data only if they agree to preserve the confidentiality of the information as requested in this form. (*Initial next to the statement if you agree)* ✱ *Required*

I understand that relevant sections of the data collected during the study, may be looked at by individuals from the University of Leeds or from

regulatory authorities where it is relevant to my taking part in this research. I give permission for these individuals to have access to my records. (*Initial next to the statement if you agree)* ✱ *Required*

I agree to take part in the above research project. (*Initial next to the statement if you agree)* ✱ *Required*

Full name (as electronic signature) ✱ *Required*

Email address ✱ *Required*

Please enter a valid email address.

Date ✱ *Required*

Dates need to be in the format 'DD/MM/YYYY', for example 27/03/1980.

(dd/mm/yyyy)

What is your role ✱ *Required*

Current practice

We would like to know what you currently do when delivering feedback on primary care prescribing issues (not just opioid medication) to general practices.

Please tick all feedback options that you currently do in practice.

- ☐ Comparison of practice achievement to top achieving practices
- ☐ Comparison of practice achievement to other practices within the same CCG
- ☐ Comparison of practice achievement to previous achievement
- ☐ Show practice achievement in a graph
- ☐ Show practice achievement in an infographic
- ☐ Show practice achievement as a number or percentage
- ☐ Show practice achievement as a combination of graph, infographic or number
- ☐ Deliver paper copies of feedback reports by post
- ☐ Deliver copies of feedback reports by email
- ☐ Deliver feedback to practices with an online dashboard (not connected to patient record system)
- ☐ Deliver feedback to practices with an online dashboard (connected to patient record system)
- ☐ Deliver feedback on total prescriptions (for a particular medication)
- ☐ Deliver feedback on total number of patients (taking a particular medication)
- ☐ Deliver feedback on specific high risk groups for a particular medication
- ☐ Give feedback on specific behaviours to change (e.g. dose reduction or particular patients to avoid prescribing for)
- ☐ Deliver educational outreach or training to practices on the feedback topic
- ☐ Ask practices to complete action plans in response to the feedback report
- ☐ Ask practices to document the potential benefits to their practice and/or patients on changing behaviour
- ☐ Provide feedback at regular intervals for the same topic (e.g. monthly, quarterly, yearly)
- ☐ Provide feedback intensely at the start of a feedback cycle and then reduce frequency
- ☐ Provide feedback for more than one year on one topic
- ☐ Provide feedback for just one year on one topic

Please comment on your current practice in using audit and feedback to primary care practices to help us with our interpretation.

Topic for primary care prescribing feedback

There are multiple high priority prescribing issues in current UK primary care.

Please score each prescribing issue identified **firstly as to its importance** (potential to improve patient safety and care) and then **secondly its priority** (based on whether there are existing interventions to improve safety and care) to tackle in an audit and feedback intervention.

Please rate the **importance** of the following prescribing issues to tackle in primary care on a scale of 1 (Low importance for further interventions) to 9 (High importance for further interventions) Click 'more info' to read more about the different proposed prescribing issues

More info

	Importance as a primary care prescribing issue									
	1	2	3	4	5	6	7	8	9	Unable to answer/do not know
Antibiotic prescribing	○	○	○	○	○	○	○	○	○	○
Opioid medication for chronic non-cancer pain	○	○	○	○	○	○	○	○	○	○
Gabapentin and pregabalin painkillers	○	○	○	○	○	○	○	○	○	○
Anticholinergic burden	○	○	○	○	○	○	○	○	○	○
Prescribing safety indicators	○	○	○	○	○	○	○	○	○	○
Prescribing in low kidney function	○	○	○	○	○	○	○	○	○	○

Please rate the **priority** to tackle in primary care of each of the following prescribing issues on a scale of 1 (Low priority for further interventions) to 9 (High priority for further interventions) Click 'more info' for more detail about the proposed prescribing issues.

More info

	Priority to tackle in an audit and feedback intervention									
	1	2	3	4	5	6	7	8	9	Unable to answer/do not know
Antibiotic prescribing	○	○	○	○	○	○	○	○	○	○
Opioid medication for chronic non-cancer pain	○	○	○	○	○	○	○	○	○	○
Gabapentin and pregabalin painkillers	○	○	○	○	○	○	○	○	○	○
Anticholinergic burden	○	○	○	○	○	○	○	○	○	○
Prescribing safety indicators	○	○	○	○	○	○	○	○	○	○
Prescribing in low kidney function	○	○	○	○	○	○	○	○	○	○

Are there any other high priority or important prescribing issues that we should add to this list?

Please comment on your scoring above to help us with our interpretation.

Audit and feedback evidence

Reviews of audit and feedback trials have shown that the effects are generally moderate or small but there may be a substantial population effect. Unfortunately, the reviews also show we still have limited information with which to support decisions on how best to use audit and feedback in routine health care.

Please rate how **useful** you feel audit and feedback to health care professionals are as methods to improve primary care prescribing on a scale of 1 (Not useful to primary care prescribers) to 9 (Extremely useful to primary care prescribers)

	1	2	3	4	5	6	7	8	9	Unable to answer/do not know
Usefulness of audit and feedback to improve primary care prescribing safety	☐	☐	☐	☐	☐	☐	☐	☐	☐	☐

Please rate how **important** you feel it is to improve the effects of audit and feedback to health care professionals on a scale of 1 (Not important) to 9 (Very important)

	1	2	3	4	5	6	7	8	9	Unable to answer/do not know
Importance of improving the effect of audit and feedback	☐	☐	☐	☐	☐	☐	☐	☐	☐	☐

Please comment on your scoring above to help us with our interpretation.

Usefulness of data

Using opioid prescribing as an example, there are different sources of primary care prescribing data that give different types of data. We would like to know which types of data would be the most useful for primary care prescribers to use to improve their opioid prescribing.

Please rate the types of data on their **usefulness** to primary care practitioners on a scale of 1 (Not at all useful) to 9 (Very useful)

	Data usefulness to primary care prescribers									
	1	2	3	4	5	6	7	8	9	Unable to answer/do not know
Total number of opioid prescriptions (Note: This includes opioid medication specifically prescribed for drug addiction (e.g. methadone) and opioid medication that may be prescribed for palliative care and cancer)	○	○	○	○	○	○	○	○	○	○
Specific opioid medications (e.g. codeine, tramadol, morphine)	○	○	○	○	○	○	○	○	○	○
Number of patients taking opioid medication	○	○	○	○	○	○	○	○	○	○
Number of patients taking opioid medication, excluding patients with a palliative care diagnosis	○	○	○	○	○	○	○	○	○	○
Number of patients taking opioid medication, excluding patients taking medication for drug addiction (silbutramine or methadone)	○	○	○	○	○	○	○	○	○	○
Sub-groups of patients (e.g. patients at high risk of escalation of opioid doses or long term opioid use or those at higher risk of adverse drug events)	○	○	○	○	○	○	○	○	○	○

If you have rated specific opioid medications as useful or important, please detail the specific medications that would be most useful and important to concentrate on.

Please comment on your scoring above to help us with our interpretation.

Randomisation

We would like to know how acceptable different levels of randomisation would be in a trial of different modifications to audit and feedback.

Please rate how **acceptable** the potential levels of randomisation for a trial on a scale of 1 (Not at all acceptable) to 9 (Very acceptable) Please click on 'more info' for further detail about primary care structure in the UK.

More info

	Acceptability of randomisation level									
	1	2	3	4	5	6	7	8	9	Unable to answer/do not know
Randomisation at practice-level										
Randomisation at practice network level										
Randomisation at Clinical Commissioning Group level										
Randomisation at Sustainability and Transformation Plan (STP) level										

Please comment on your scoring above to help us with our interpretation.

Consent

There are ethical decisions relating to consent to taking part in a trial of different modifications to audit and feedback on opioid prescribing in primary care, e.g. consent for practice-aggregated data extraction from medical records and consenting to taking part in a randomised experiment. Please rate the following different consent options on **firstly, how acceptable they are** and **secondly how ideal** they are.

Please rate how **acceptable** the potential options for consent for a trial on a scale of 1 (Not at all acceptable) to 9 (Very acceptable)

More info

	Acceptability of randomisation level									
	1	2	3	4	5	6	7	8	9	Unable to answer/do not know
Consent practices individually, asking them to sign up to a opioid prescribing feedback trial (practice opt-in)	○	○	○	○	○	○	○	○	○	○
Provide practices information on the trial and allow them to withdraw from the trial if they wish (practice opt-out – likely to increase recruitment compared to opt-in)	○	○	○	○	○	○	○	○	○	○
Consent at Clinical Commissioning Group level for data access	○	○	○	○	○	○	○	○	○	○
Consent at Sustainablity and Transformation Plan level for data access	○	○	○	○	○	○	○	○	○	○
Waive consent as the burden of responding to consent request is higher than taking part in the trial (used by NHS England feedback study on antibiotic prescribing)	○	○	○	○	○	○	○	○	○	○

Please rate how **ideal** the potential options for consent for a trial on a scale of 1 (Not at all ideal) to 9 (Very ideal)

More info

	Ideal randomisation level									
	1	2	3	4	5	6	7	8	9	Unable to answer/do not know
Consent practices individually, asking them to sign up to a opioid prescribing feedback trial (practice opt-in)	○	○	○	○	○	○	○	○	○	○
Provide practices information on the trial and allow them to withdraw from the trial if they wish (practice opt-out – likely to increase recruitment compared to opt-in)	○	○	○	○	○	○	○	○	○	○
Consent at Clinical Commissioning Group level for data access	○	○	○	○	○	○	○	○	○	○
Consent at Sustainablity and Transformation Plan level for data access	○	○	○	○	○	○	○	○	○	○

Waive consent as the burden of responding to consent request is higher than taking part in the trial (used by NHS England feedback study on antibiotic prescribing)	○	○	○	○	○	○	○	○	○	○

Please comment on your scoring above to help us with our interpretation.

Report delivery

We would like to know how interested you are in finding out whether the following potential **methods of delivery of feedback reports** to primary care practices are the **most effective** at improving prescribing.

Please rate each item **firstly on its acceptability** to practices and then **secondly on how ideal** it is for practices.

Please rate how **acceptable** the potential options for feedback delivery are on a scale of 1 (Not at all acceptable) to 9 (Very acceptable)

	Acceptablility of feedback delivery methods									
	1	2	3	4	5	6	7	8	9	Unable to answer/do not know
Provide (multiple) copies of a paper-based feedback report to each practice	○	○	○	○	○	○	○	○	○	○
Send a PDF copy of the report via email to each practice	○	○	○	○	○	○	○	○	○	○
Have an online dashboard that practices can log into to view their report (not linked to patient record system)	○	○	○	○	○	○	○	○	○	○
Have an online dashboard that practices can log into that connects to the patient record system to identify patients where review is needed	○	○	○	○	○	○	○	○	○	○

Please rate how **ideal** the potential options for feedback delivery are on a scale of 1 (Not at all ideal) to 9 (Very ideal)

	Ideal feedback delivery methods									
	1	2	3	4	5	6	7	8	9	Unable to answer/do not know
Provide (multiple) copies of a paper-based feedback report to each practice	○	○	○	○	○	○	○	○	○	○
Send a PDF copy of the report via email to each practice	○	○	○	○	○	○	○	○	○	○
Have an online dashboard that practices can log into to view their report (not linked to patient record system)	○	○	○	○	○	○	○	○	○	○
Have an online dashboard that practices can log into that connects to the patient record system to identify patients where review is needed	○	○	○	○	○	○	○	○	○	○

Please comment on your scoring above to help us with our interpretation.

Modifications to feedback reports

Even on the basis of the best evidence available, no strong recommendations can be given regarding the best way to introduce audit and feedback into routine practice. We wish to test important (but potentially subtle) variations in audit and feedback that may have important effects.

We would like to know how interested you are in finding out whether the following potential modifications to feedback reports are the most effective.

Please rate how **interested** you would be in finding out if the following options for feedback modifications are **the most effective** are on a scale of 1 (Not at all interested) to 9 (Very interested)

	Interest in finding out if feedback modification is more effective									
	1	2	3	4	5	6	7	8	9	Unable to answer/do not know
Whether different comparators within the reports are more effective (e.g. comparison to top achieving practices or comparison to practices within same geographical area or comparison to previous achievement)	○	○	○	○	○	○	○	○	○	○
Whether different visual interpretations of the data are more effective (e.g. bar chart of all practices in geographical area or an infographic of achievement or a number stating achievement)	○	○	○	○	○	○	○	○	○	○
Whether different delivery methods of providing feedback are more effective (e.g. by post, email or an online dashboard)	○	○	○	○	○	○	○	○	○	○
Whether feedback about individual or aggregated cases is more effective (e.g. total opioid prescriptions or number of patients taking opioids)	○	○	○	○	○	○	○	○	○	○
Whether feedback identifying specific behaviours to be changed is more effective (e.g. reducing opioid drug dose escalation, reduction in patients taking combinations of opioids)	○	○	○	○	○	○	○	○	○	○
Whether feedback on its own is more (cost-) effective than feedback delivered with educational outreach or training	○	○	○	○	○	○	○	○	○	○
Whether asking practitioners to document the implications of changing practice is more effective (e.g. asking practitioners to document the potential benefits for their patients or their practice by reducing opioid prescriptions)	○	○	○	○	○	○	○	○	○	○

Whether the frequency or the number of times feedback is delivered affects achievement (e.g. providing quarterly feedback for 1, 2 or 3 years or whether an increased frequency (monthly) at the start of the feedback period followed by a reduction in frequency (to 6 monthly) after 1 year is more effective than regular quarterly feedback)	○	○	○	○	○	○	○	○	○	○

Are there any other high priority or important A&F modifications that we should add to this list for testing?

Please comment on your scoring above to help us with our interpretation.

Final page

Thank you for completing this consensus questionnaire, I am very grateful for your time. I will be collating all responses and will send you the second round of this consensus questionnaire in late June with a copy of your answers and all participant answers combined. You will then have a chance to re-answer the questions based on the results.

If you have any comments regarding this questionnaire or the consensus process please email Sarah Alderson (s.l.alderson@leeds.ac.uk).

Key for selection options

11 - What is your role

Audit and Feedback Researcher
Medicines Optimisation Lead
Patient and Public Representative

Figure S1: Medicines Optimisation Lead's (n=5) current prescribing feedback practice

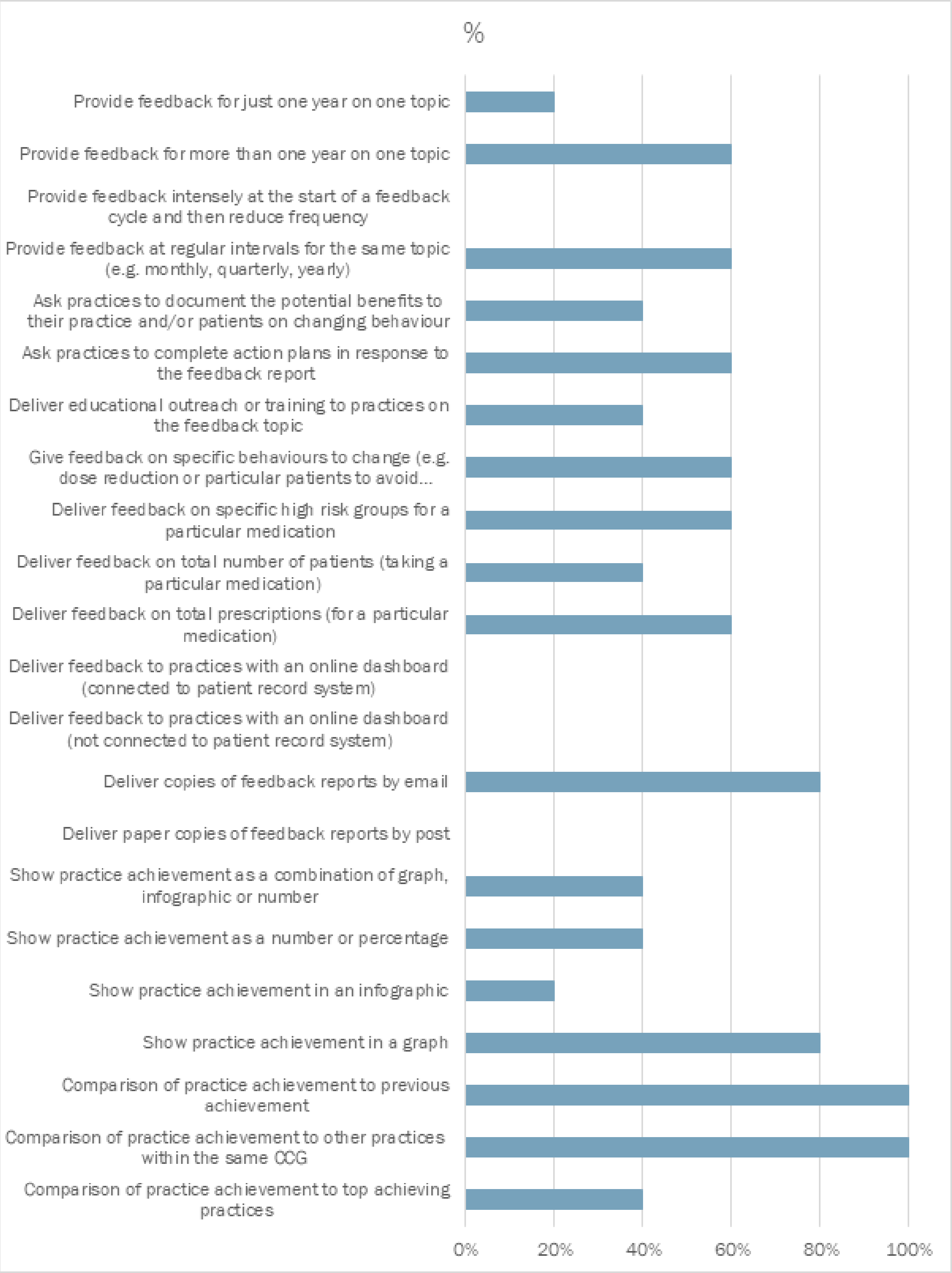

Table S1

Recommendations for a primary care prescribing implementation laboratory

No.	Recommendation	A&F Researcher's (n=5) score (1-9)	Medicine Optimisation Lead's (n=5) score (1-9)	Patient & Public Involvement & Engagement's (n=4) score (1-9)	% Consensus (n=14)
Prescribing issues for A&F:					
- Importance					
1.	Antibiotic prescribing	9	9	8.5	93
2.	Prescribing safety indicators	7	8	7.5	93
3.	Opioid medication for chronic, non-cancer pain	9	9	7	86
4.	Anticholinergic burden	6	8	7	79
5.	Prescribing in low kidney function	8	7	6.5	72
6.	Gabapentin and pregabalin painkillers	5	8	6	72
- Priority					
7.	Antibiotic prescribing	9	9	8.5	100
8.	Opioid medication for chronic, non-cancer pain	9	8.5	7.5	93
9.	Anticholinergic burden	7.5	7	7	86
10.	Prescribing safety indicators	7.5	7	7.5	79
11.	Prescribing in low kidney function	7.5	6	6.5	72
	Gabapentin and pregabalin painkillers	*6.5*	*9*	*8*	*67*
Audit and feedback as a method to improve primary care prescribing					
12.	Importance	8	8	9	93
13.	Usefulness	8	8	7	86
Usefulness of types of data					
14.	Sub-groups of patients at high risk of dose escalation or adverse effects	8	9	9	100
15.	Number of patients taking opioid medication, excluding patients with a palliative care diagnosis	6.5	8	7.5	86
16.	Number of patients taking opioid medication, excluding patients taking medication for drug addiction	6	8	8	73
	Specific opioid medications	*7*	*7*	*7*	*50*
	Number of patients taking opioid medication	*5*	*6*	*7.5*	*50*
	*Total number of opioid prescriptions***	*3*	*3*	*3.5*	*7*
Randomisation level					
17.	Randomisation at practice level	9	9	9	100
18.	Randomisation at primary care network level	9	9	8	91
19.	Randomisation at Clinical Commissioning Group level	8	8	7.5	75

	Randomisation at Sustainability and Transformation Plan level	*9*	*7*	*8*	*63*
Consent					
- Acceptable					
20.	Provide practices information on the trial and allow them to withdraw from the trial if they wish (practice opt-out)	9	9	9	100
	Consent at Clinical Commissioning Group level for data access	*6*	*8*	*7.5*	*68*
	Waive consent as the burden of responding to consent request is higher than taking part in the trial	*7*	*6*	*8*	*62*
	Consent practices individually, asking them to sign up to an opioid prescribing feedback trial (practice opt-in)	*7*	*5*	*6.5*	*36*
	Consent at Sustainability and Transformation Plan level for data access	*6*	*6*	*6*	*36*
- Ideal					
21.	Provide practices information on the trial and allow them to withdraw from the trial if they wish (practice opt-out)	8	8	8	86
22.	Waive consent as the burden of responding to consent request is higher than taking part in the trial	8	9	8	77
23.	Consent at Clinical Commissioning Group level for data access	7	8	4.5	77
	Consent at Sustainability and Transformation Plan level for data access	*6*	*7*	*6.5*	*64*
	Consent practices individually, asking them to sign up to an opioid prescribing feedback trial (practice opt-in)	*5*	*2*	*5.5*	*14*
Feedback delivery method					
- Acceptable					
24.	Have an online dashboard that practices can log into that connects to the EHR to identify patients where review is needed	9	9	9	100
	Have an online dashboard that practices can log into to view their report (not linked to EHR system)	*7*	*7*	*7.5*	*64*
	Send a PDF copy of the report via email to each practice	*6*	*6*	*6.5*	*43*
	Provide (multiple) copies of a paper-based report to each practice	*6*	*3*	*4*	*21*
- Ideal					
25.	Have an online dashboard that practices can log into that connects to the EHR to identify patients where review is needed	9	9	9	100
26.	Have an online dashboard that practices can log into to view their report (not linked to EHR system)	8	7	7	79
	Send a PDF copy of the report via email to each practice	*5*	*5*	*5.5*	*7*
	*Provide (multiple) copies of a paper-based report to each practice***	*3*	*1*	*2.5*	*2*
Feedback modifications to test for effectiveness					
27.	Whether feedback identifying specific behaviours to be changed is more effective	8	8	7.5	93
28.	Whether different comparators within the reports are more effective	8	8	7	86
29.	Whether feedback about individual or aggregated cases is more effective	7	9	7.5	79

30.	Whether the frequency or the number of times feedback is delivered affects achievement	6	7	7	79
31.	Whether different visual interpretations of the data are more effective	7	9	7.5	71
32.	Whether feedback on its own is more (cost-) effective than feedback delivered with educational outreach or training	8	8	7	71
33.	Whether different delivery methods of providing feedback are more effective	7	7	8	71
	Whether asking practitioners to document the implications of changing practice is more effective	*6*	*7*	*5.5*	*43*
Involved in designing feedback reports					
34.	General Practitioners	8	9	9	100
35.	Primary Care Pharmacists	8	8	9	93
36.	Medicine Optimisation Leads	8	9	8.5	92
	Clinical Commissioners	*6*	*6*	*7*	*46*
	Patient and Public Involvement Experts	*5*	*6*	*6*	*36*

*Italicised text indicates areas not reaching consensus; ** indicates areas reaching consensus not for inclusion*
