# Supplementary figures and images for: Establishing a primary care audit and feedback implementation laboratory: a consensus study
Source: Implement Sci Commun. 2021 Jan 7;2:3. doi: 10.1186/s43058-020-00103-8 (PMC7792204; doi:10.1186/s43058-020-00103-8)

Appendix 2: Medicines Optimisation Lead’s (n=5) current prescribing feedback practice


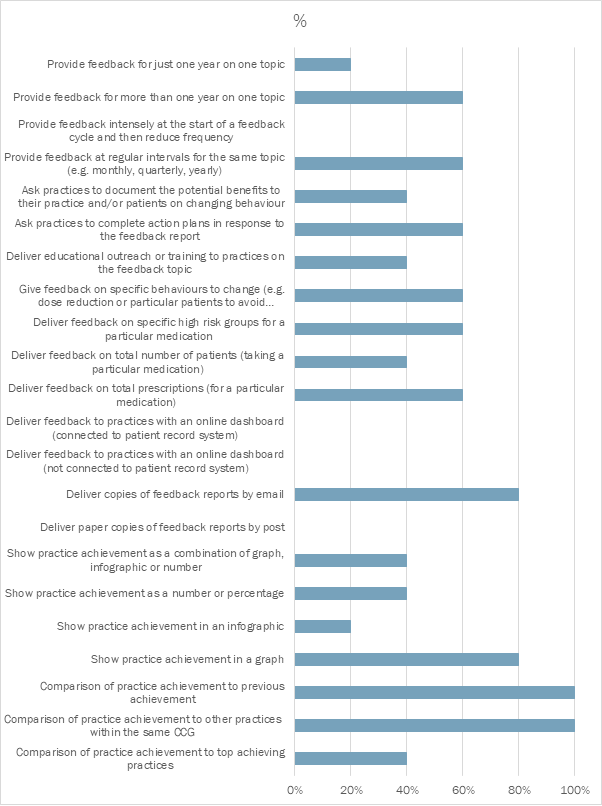

Supplement: Supplementary file 2 — Additional file 2. Medicines Optimisation Lead’s (n = 5) current prescribing feedback practice [file 43058_2020_103_MOESM2_ESM.docx]
