# Supplementary material for: Establishing a primary care audit and feedback implementation laboratory: a consensus study
Source: Implement Sci Commun. 2021 Jan 7;2:3. doi: 10.1186/s43058-020-00103-8 (PMC7792204; doi:10.1186/s43058-020-00103-8)
Supplement: Supplementary file 3 — Additional file 3. Results from the first round of consensus survey. Results that changed in round 2 are in italics and bold [file 43058_2020_103_MOESM3_ESM.docx]

# Appendix 3

Results from the first round of consensus survey. Results that changed in round 2 are in italics and bold.

| **No.** | **Recommendation** | **A&F Researcher’s (n=5) score (1-9)** | **Medicine Optimisation Lead’s (n=5) score (1-9)** | **Patient & Public Involvement & Engagement’s (n=4) score (1-9)** | **% Consensus (n=14)** |
| --- | --- | --- | --- | --- | --- |
| Prescribing issues for A&F: | | | | | |
| - Importance | | | | | |
| 1. | Antibiotic prescribing | 9 | 9 | ***5*** | ***75*** |
| 2. | Prescribing safety indicators | ***6*** | ***7.5*** | ***7*** | ***73*** |
| 3. | Opioid medication for chronic, non-cancer pain | ***7*** | 9 | ***7*** | **67** |
| 4. | Anticholinergic burden | ***5.5*** | 8 | 7 | ***56*** |
| 5. | Prescribing in low kidney function | 8 | 7 | ***6*** | ***73*** |
| 6. | Gabapentin and pregabalin painkillers | 5 | 8 | ***5*** | ***60*** |
| - Priority | | | | | |
| 7. | Antibiotic prescribing | ***8*** | 9 | 8.5 | ***83*** |
| 8. | Opioid medication for chronic, non-cancer pain | 9 | ***9*** | ***7.5*** | ***83*** |
| 9. | Anticholinergic burden | 7.5 | 7 | ***7.5*** | ***67*** |
| 10. | Prescribing safety indicators | 7.5 | 7 | ***7*** | ***64*** |
| 11. | Prescribing in low kidney function | 7.5 | 6 | ***6*** | ***64*** |
|  | Gabapentin and pregabalin painkillers | ***5.5*** | 9 | ***7*** | ***70*** |
| Audit and feedback as a method to improve primary care prescribing | | | | | |
| 12. | Importance | 8 | 8 | ***7*** | ***86*** |
| 13. | Usefulness | 8 | 8 | ***9*** | ***100*** |
| Usefulness of types of data | | | | | |
| 14. | Sub-groups of patients at high risk of dose escalation or adverse effects | 8 | 9 | 9 | 100 |
| 15. | Number of patients taking opioid medication, excluding patients with a palliative care diagnosis | 6.5 | 8 | ***9*** | ***83*** |
| 16. | Number of patients taking opioid medication, excluding patients taking medication for drug addiction | 6 | 8 | ***9*** | 73 |
|  | Specific opioid medications | 7 | 7 | ***7.5*** | ***67*** |
|  | Number of patients taking opioid medication | ***3*** | 6 | 7.5 | ***54*** |
|  | Total number of opioid prescriptions** | ***2*** | 3 | 3.5 | ***23*** |
| Randomisation level | | | | | |
| 17. | Randomisation at practice level | 9 | 9 | ***7*** | ***85*** |
| 18. | Randomisation at primary care network level | 9 | 9 | ***4*** | ***67*** |
| 19. | Randomisation at Clinical Commissioning Group level | ***7.5*** | 8 | ***3*** | ***64*** |
|  | Randomisation at Sustainability and Transformation Plan level | 9 | 7 | ***6*** | 63 |
| Consent | | | | | |
| - Acceptable | | | | | |
| 20. | Provide practices information on the trial and allow them to withdraw from the trial if they wish (practice opt-out) | 9 | 9 | 9 | ***86*** |
|  | Consent at Clinical Commissioning Group level for data access | 6 | 8 | ***4*** | ***50*** |
|  | Waive consent as the burden of responding to consent request is higher than taking part in the trial | 7 | ***7.5*** | ***5*** | 62 |
|  | Consent practices individually, asking them to sign up to an opioid prescribing feedback trial (practice opt-in) | ***9*** | ***6*** | ***5*** | 36 |
|  | Consent at Sustainability and Transformation Plan level for data access | ***8.5*** | ***3.5*** | ***2*** | ***44*** |
| - Ideal | | | | | |
| 21. | Provide practices information on the trial and allow them to withdraw from the trial if they wish (practice opt-out) | 8 | 8 | ***7.5*** | ***79*** |
| 22. | Waive consent as the burden of responding to consent request is higher than taking part in the trial | ***6.5*** | ***3*** | 8 | ***69*** |
| 23. | Consent at Clinical Commissioning Group level for data access | 7 | 8 | 4.5 | ***62*** |
|  | Consent at Sustainability and Transformation Plan level for data access | ***8*** | ***6.5*** | ***4*** | ***50*** |
|  | Consent practices individually, asking them to sign up to an opioid prescribing feedback trial (practice opt-in) | ***7*** | 2 | ***2.5*** | ***36*** |
| Feedback delivery method | | | | | |
| - Acceptable | | | | | |
| 24. | Have an online dashboard that practices can log into that connects to the EHR to identify patients where review is needed | 9 | 9 | ***8.5*** | ***93*** |
|  | Have an online dashboard that practices can log into to view their report (not linked to EHR system) | 7 | 7 | ***5.5*** | ***57*** |
|  | Send a PDF copy of the report via email to each practice | 6 | 6 | ***5*** | 43 |
|  | Provide (multiple) copies of a paper-based report to each practice | 6 | 3 | ***3*** | 21 |
| - Ideal | | | | | |
| 25. | Have an online dashboard that practices can log into that connects to the EHR to identify patients where review is needed | 9 | 9 | ***8.5*** | ***93*** |
| 26. | Have an online dashboard that practices can log into to view their report (not linked to EHR system) | 8 | 7 | ***5.5*** | ***64*** |
|  | Send a PDF copy of the report via email to each practice | 5 | 5 | ***4.5*** | ***14*** |
|  | Provide (multiple) copies of a paper-based report to each practice** | ***6*** | 1 | ***3.5*** | ***21*** |
| Feedback modifications to test for effectiveness | | | | | |
| 27. | Whether feedback identifying specific behaviours to be changed is more effective | 8 | 8 | ***8*** | ***77*** |
| 28. | Whether different comparators within the reports are more effective | 8 | 8 | ***5.5*** | ***79*** |
| 29. | Whether feedback about individual or aggregated cases is more effective | 7 | 9 | ***8*** | ***57*** |
| 30. | Whether the frequency or the number of times feedback is delivered affects achievement | 6 | 7 | ***5.5*** | ***57*** |
| 31. | Whether different visual interpretations of the data are more effective | ***6*** | 9 | ***6*** | ***64*** |
| 32. | Whether feedback on its own is more (cost-) effective than feedback delivered with educational outreach or training | 8 | 8 | ***6*** | ***77*** |
| 33. | Whether different delivery methods of providing feedback are more effective | 7 | ***6*** | 8 | ***57*** |
|  | Whether asking practitioners to document the implications of changing practice is more effective | 6 | 7 | ***4.5*** | 43 |
| Involved in designing feedback reports | | | | | |
| 34. | General Practitioners | 8 | 9 | ***7.5*** | ***86*** |
| 35. | Primary Care Pharmacists | 8 | ***9*** | ***7*** | ***79*** |
| 36. | Medicine Optimisation Leads | 8 | 9 | ***5.5*** | ***82*** |
|  | Clinical Commissioners | ***7*** | 6 | 7 | ***50*** |
|  | Patient and Public Involvement Experts | 5 | 6 | ***5.5*** | 36 |
